# Closing/Closed Gastroschisis (CGS): Antenatal Predictors and Surgical Strategies in Cases of Unique Anatomy from a Case Series

**DOI:** 10.3390/children13030408

**Published:** 2026-03-15

**Authors:** Dmitrii Morozov, Liza Vanyan, Mariia Morozova, Nadezhda Erokhina, Ellina Velichko, Olga Morozova, Maria Yagodkina, Vasily Shumikhin, Olga Mokrushina

**Affiliations:** 1 Department of Pediatric Surgery, Institute of Motherhood and Childhood, Pirogov Russian National Research Medical University, 1, Ostrovityanova Street, 117997 Moscow, Russia; MorozovDD@zdrav.mos.ru (D.M.); lizzzzka2019@mail.ru (L.V.); m.belyakova02@mail.ru (M.M.); shumikhinvs@zdrav.mos.ru (V.S.); MokrushinaOG@zdrav.mos.ru (O.M.); 2 Department of Newborn and Premature Babies Surgery, Filatov Children’s City Clinical Hospital, 15, Sadovaya-Kudrinskaya Street, 123001 Moscow, Russia; ErokhinaNO1@zdrav.mos.ru; 3 Department of Pathophysiology, Institute of Digital Biodesign and Artificial Intelligence in Medicine, I.M. Sechenov First Moscow State Medical University (Sechenov University), 119019 Moscow, Russia; velichko_e_v@staff.sechenov.ru; 4 Clinical Pathophysiology Laboratory, Veltischev Research and Clinical Institute for Pediatrics and Pediatric Surgery, Pirogov Russian National Research Medical University, 2, Taldomskaya Street, 125412 Moscow, Russia; 5 Department of Antenatal Fetal Care, Yudin City Clinical Hospital, 4, Kolomensky Proezd, 101000 Moscow, Russia; YagodkinaMA@zdrav.mos.ru

**Keywords:** closed gastroschisis, closing gastroschisis, complex gastroschisis, short-bowel syndrome, “vanishing bowel”

## Abstract

**Highlights:**

**What are the main findings?**

• The appearance of sonographic signs of intestinal obstruction in the second trimester may be a predictor for a high risk of subsequent significant vascular compromise of the eviscerated bowel, potentially leading to the more severe types of CGS (C and D).
• For patients with CGS type B, a staged surgical approach is advisable to maximize bowel length preservation.

**What are the implications of the main findings?**

• This research could enable the development of clear guidelines for intensified ultrasound screening, timing of early delivery, and even the consideration of fetal surgery to prevent extensive intestinal loss.
• Improving outcomes for patients with closing/closed gastroschisis by preserving the entire gastrointestinal tract

**Abstract:**

**Background**: Closing/closed gastroschisis (CGS) accounts for approximately 6% of gastroschisis cases globally. Currently, no consensus exists regarding: antenatal predictors of CGS types, optimal antenatal management (ultrasound screening frequency, indications for early delivery), or standardized surgical strategies tailored to CGS type (staging/timing of procedures, enterostomy necessity/level). **Methods**: Five neonates with CGS were enrolled and classified according to Perrone’s classification: two patients with type B (40%), one with type C (20%), one with type D (20%), one patient was classified as unclear (20%). Gender distribution—80% female (*n* = 4), 20% male (*n* = 1); gestational age—median 35 weeks (IQR 35–38); preterm birth rate—80% (*n* = 4); birth weight—median 2620 g (IQR 2310–3850). **Results**: Three patients (60%) developed antenatal intestinal obstruction signs at the third trimester, including two who postnatally demonstrated viable intestinal loops. Two patients (40%) with necrosis of eviscerated intestine demonstrated onset of antenatal intestinal obstruction signs at the second trimester. Patients with CGS type B were managed using a staged surgical approach; patients with types C and D received single-stage repair. Patient with CGS type B achieved complete clinical recovery. Three patients (60%) with CGS types C and D developed short bowel syndrome. **Conclusions**: The appearance of sonographic signs of intestinal obstruction in the second trimester may be a predictor for a high risk of subsequent significant vascular compromise of the eviscerated bowel, leading to more severe types of CGS (C and D). For patients with CGS type B, a staged surgical approach is advisable to maximize bowel length preservation.

## 1. Introduction

Gastroschisis (GS) is a congenital anomaly of the abdominal wall, characterized by the evisceration of abdominal contents through a full-thickness defect typically located to the right of the umbilical cord insertion [1]. Prenatal ultrasound typically detects this condition, which has a global incidence ranging from 2 to 5 cases per 10,000 live births [2]. Most cases are classified as simple gastroschisis, which correlates with minimal morbidity and excellent survival rates [3]. The postoperative care for these newborns is largely standardized and universally accepted within the pediatric surgical community. However, approximately 11–17% of cases are designated as complex, involving complications such as intestinal atresia, stenosis, perforation, necrosis, or volvulus. These comorbidities complicate surgical decision-making due to compromised intestinal viability and often portend a poor prognosis [4,5,6]. Patients in this complex group face an elevated risk of extended hospitalization, short bowel syndrome, and death [7,8]. Closing/closed gastroschisis (CGS) represents a particularly severe form of complex gastroschisis, characterized by constriction of the eviscerated bowel at the level of the fascial defect [9,10]. The etiology and pathogenesis of CGS remain unclear, and the potential for accurate antenatal diagnosis continues to represent a significant unresolved question [1,11]. Prenatal strangulation of the intestines and mesentery occurs secondary to progressive constriction or complete closure of the abdominal wall defect. This results in partial or complete vascular compromise of the eviscerated bowel, leading to necrosis or even complete “vanishing” of the affected segments. CGS accounts for approximately 6% of GS cases globally [12]. Consequently, short bowel syndrome (SBS) develops postnatally in 2–27% of cases [13,14]. Data on mortality associated with CGS are limited due to the rarity of this condition, with the literature largely consisting of isolated case reports and small case series. Nevertheless, contemporary estimates of mortality for this form of complex gastroschisis range from 16.7% to as high as 70–83% [15,16].

An important issue is the absence of a standardized classification for CGS among researchers, which poses a considerable obstacle to conducting meaningful systematic reviews and data synthesis on this uncommon condition. Perrone and colleagues (2019) developed a classification system based on the observation that patients with CGS represent a spectrum of disease with varying degrees of bowel loss and complications, rather than a single, uniform diagnosis: A = bowel that is significantly constricted at the ring with ischemia but without atresia; Type B = bowel that is significantly constricted at the ring with ischemia and associated atresia; Type C = presence of a closing ring with non-viable external bowel ± atresia; Type D = completely closed defect with either a nubbin of exposed tissue or no external bowel [10]. However, this classification has not yet been widely adopted or correlated with antenatal findings. Given the rarity of CGS at individual centers, detailed case series employing standardized classifications are essential to enhance collective knowledge and optimize patient management.

Currently, no consensus exists regarding: antenatal predictors of CGS types; optimal antenatal management (e.g., ultrasound screening frequency, indications for early delivery); standardized surgical strategies tailored to CGS type (e.g., staging/timing of procedures, enterostomy necessity/level) [5,10].

Between 2023 and 2024, our clinic admitted 24 neonates with gastroschisis, of whom 5 (20.8%) presented with CGS. The rising incidence of CGS, coupled with its high mortality and poor prognoses, underscores the urgent need to investigate etiopathogenesis and validate antenatal predictors for severe subtypes (types C and D/”vanishing bowel”).

In this study, we present a case series of patients with CGS, utilizing the current classification system (Perrone’s classification) to analyze potential antenatal predictors of specific types and characterize the unique anatomical variations that necessitate tailored surgical approaches. The work is aimed to show the feasibility of antenatal diagnosis of closing/closed gastroschisis.

## 2. Materials and Methods

The inclusion criteria comprised: 1. Antenatal diagnosis of gastroschisis, 2. Postnatal confirmation of either a narrow abdominal wall defect (<2 cm diameter) or complete absence of the defect.

Between 2023 and 2024, five neonates meeting these criteria were enrolled and classified according to the Perrone’s classification. Accordingly, there were 2 patients with type B (40%), 1 with type C (20%), 1 with type D (20%), and 1 patient was classified as unclear (20%). The cohort demonstrated the following demographic characteristics: Gender distribution: 80% female (*n* = 4), 20% male (*n* = 1); gestational age: median 35 weeks (IQR 35–38); preterm birth rate: 80% (*n* = 4); birth weight: median 2620 g (IQR 2310–3850).

## 3. Results

### 3.1. Case 1

**Figure 1:**
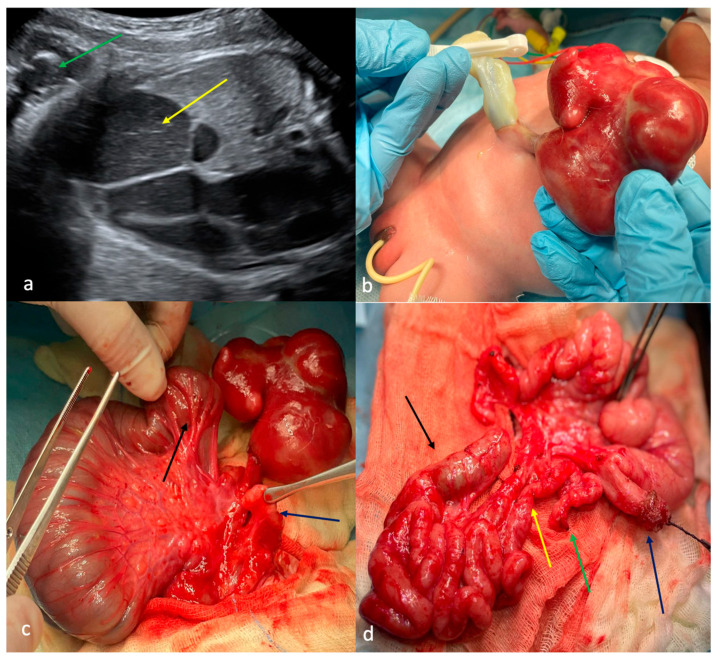
Case 1 with CGS type B. (**a**)—ultrasound screening at 31 weeks of gestation: intestinal loops into the amniotic cavity (green arrow), dilated intraabdominal intestinal loops (yellow arrow); (**b**)—appearance: narrow defect in the anterior abdominal wall to the left of the umbilical ring, the small bowel loop complex enclosed in dense fibrin; (**c**)—first operation on the first day of life: jejunal atresia (black arrow), mesentery with the vessel leading to the eviscerated bowel loop complex, “microcolon” (dark blue arrow); (**d**)—relaporotomy at the age of 44 days: earlier eviscerated bowel loop complex including loops of the jejunum and ileum, the caecum with the appendix, and part of the ascending colon that ended blindly (black arrow), jejunum stenosis (yellow arrow), distal end of jejunum atresia (green arrow), proximal end of jejunum atresia—jejunostoma (dark blue arrow).

First-trimester ultrasound screening identified gastroschisis. Antenatal signs of intestinal obstruction (dilated intestinal loops with pendular peristalsis, polyhydramnios) were detected at 31 weeks of gestation with satisfactory intramural blood supply of eviscerated non-dilated intestinal loops (Figure 1a). The patient presented with a narrow paraumbilical defect of the anterior abdominal wall on the left side and a small eviscerated complex of empty intestinal loops encased in dense fibrin with preserved bowel vascularization (CGS type B, Figure 1b). There were ultrasonographic signs of congenital lower intestinal obstruction without evidence of peritonitis and with no associated congenital defects.

Surgical treatment consisted of a two-stage surgical approach. The first surgical step was performed within the first 24 h of life: the proximal jejunal atresia with colonic atresia and microcolon were exposed and the excised bowel loop complex was rerouted into the abdominal cavity, a jejunostomy was created at the level of the jejunal atresia, and the abdominal wall defect was primarily closed (Figure 1c). The second surgical step was performed on the 44th day of life to restore intestinal continuity: the satisfactory viability of the previously eviscerated intestinal loops, with jejunal stenosis at the site of previous abdominal wall compression with a blind-ending jejunal segment 3 cm proximal to the stenosis, was revealed, which confirmed associated type IIIA jejunal atresia (Figure 1d). Surgical treatment included a jejunojejunal anastomosis, a colocolic anastomosis, and a Heineke–Mikulicz jejunoplasty of the 3 cm stenotic segment. The patient maintained full intestinal length and achieved complete clinical recovery without postoperative complications.

### 3.2. Case 2

**Figure 2:**
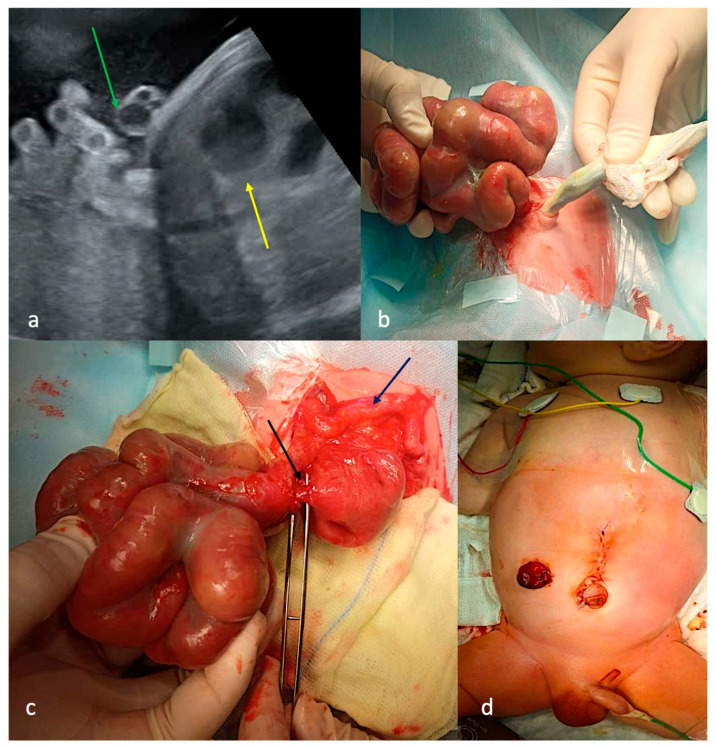
Case 2 with CGS type B. (**a**)—ultrasound screening at 31 weeks of gestation: intestinal loops into the amniotic cavity (green arrow), dilated intraabdominal intestinal loops (yellow arrow); (**b**)—appearance: narrow defect of the anterior abdominal wall to the right of the umbilical ring, through which intestinal loops were eviscerated and filled with contents; (**c**)—first operation on the first day of life: jejunum stenosis (black arrow), microcolon (dark blue arrow); (**d**)—appearance after immersion of the bowel loop complex, abdominoplasty and jejunostomy.

First-trimester ultrasound screening identified gastroschisis. Antenatal signs of intestinal obstruction were detected at 31 weeks of gestation with a preserved intramural blood supply of eviscerated dilated intestinal loops (Figure 2a). The patient presented with a narrow paraumbilical defect of the anterior abdominal wall on the right side and an eviscerated complex of dilated, content-filled intestinal loops encased in dense fibrin with preserved bowel vascularization (CGS type B, Figure 2b). The patient underwent a three-stage surgical approach. The first surgical step was performed within the first 24 h of life: a jejunal stenosis at the junction with the eviscerated intestinal loops with distal ileal atresia was diagnosed and the eviscerated bowel loop complex was repositioned into the abdominal cavity with the creation of a stoma at the level of the jejunal stenosis and primary closure of the abdominal wall defect (Figure 2c). The second surgical step was performed on the 32nd day of life: the procedure was limited to jejunojejunal anastomosis with ileostomy formation due to impaired peristalsis and wall thickening of the previously eviscerated loops (Figure 2d). At three months of age, the patient subsequently underwent end-to-side ileocolic anastomosis as the final reconstructive stage. After the third surgical treatment, the patient developed septic shock with subsequent multi-organ failure, ultimately resulting in mortality.

### 3.3. Case 3

**Figure 3:**
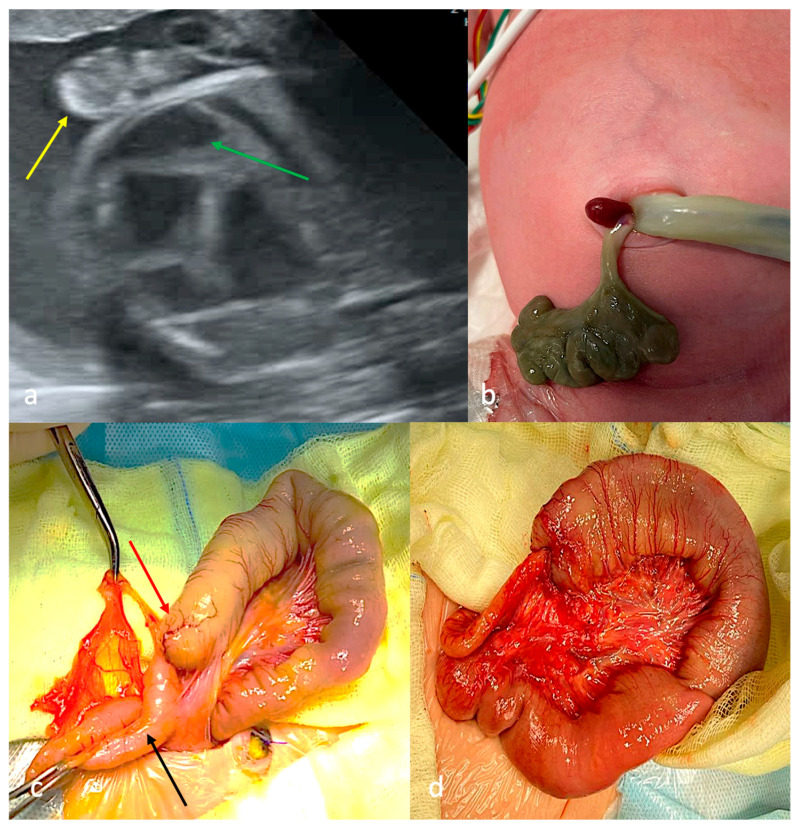
Case 3 with CGS type C. (**a**)—ultrasound screening at 33 weeks of gestation: intestinal loops into the amniotic cavity without blood supply (yellow arrow), dilated intraabdominal intestinal loops (green arrow); (**b**)—appearance: defect in the anterior abdominal wall, paraumbilically on the right side, through which small volumes of non-viable intestinal loops were eviscerated and connected to the abdominal cavity via a connective tissue cord; (**c**)—operation on the first day of life: jejunal atresia (red arrow), colon atresia (black arrow); (**d**)—jejuno-colonanastomosis.

Ultrasound examination in the first trimester revealed gastroschisis and reduced blood flow to the eviscerated intestines. Signs of intestinal obstruction were noted at 19 weeks of gestation, and at 27 weeks of pregnancy there was no blood flow in the eviscerated intestine (Figure 3a). The patient presented with non-viable eviscerated intestinal loops connected to the abdominal cavity via a connective tissue cord (Figure 3b). There were ultrasonographic signs of congenital lower intestinal obstruction without evidence of peritonitis and with no associated congenital defects. Surgery was performed within the first 24 h of life. Initial surgical exploration revealed proximal jejunal atresia with colonic atresia and microcolon; jejunocolic anastomosis was performed (Figure 3c,d). There were no postoperative complications, but the patient subsequently had to be treated in the gastroenterology department due to short bowel syndrome (SBS).

### 3.4. Case 4

**Figure 4:**
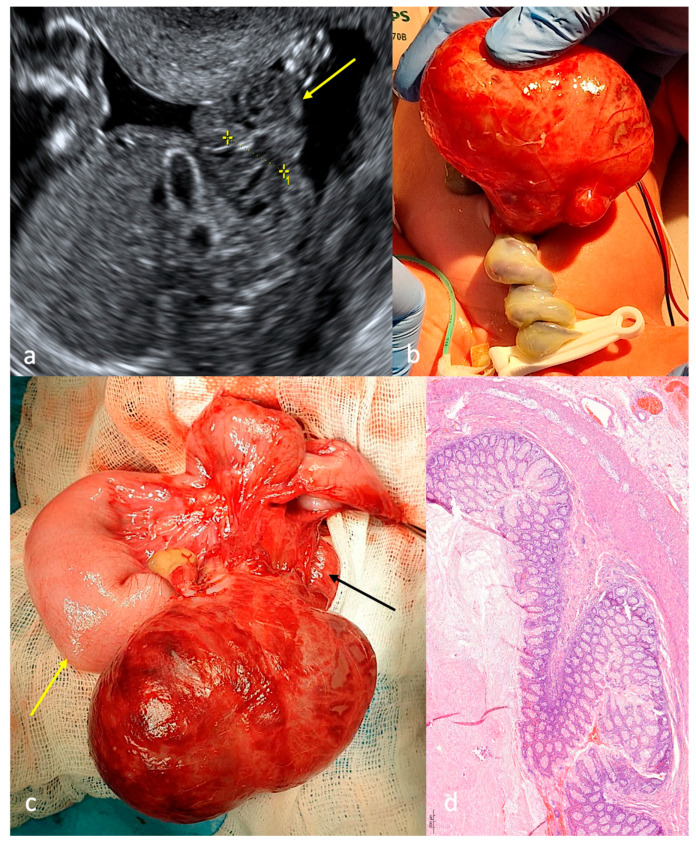
Case 4 with CGS (type C?). (**a**)—ultrasound screening at 33 weeks of gestation: intestinal loops into the amniotic cavity without blood supply (yellow arrow), “1”—anterior abdominal wall defect; (**b**)—appearance after birth: small defect of the anterior abdominal wall up to the umbilical cord, through which a soft-elastic cystic formation was eviscerated; (**c**)—operation on the first day of life: jejunal atresia (yellow arrow), colon atresia (black arrow); (**d**)—morphological structure of the cystic formation wall: structure of the colon, including all its layers (magnification ×200).

First-trimester ultrasound screening identified gastroschisis. Antenatal signs of intestinal obstruction were detected at 32 weeks of gestation (Figure 4a). Notably, the fetus presented a 44-mm cystic mass containing anechoic fluid in the amniotic cavity at 35 weeks of gestation. The patient presented with a soft, elastic cystic structure with thin walls that had eviscerated through a narrow paraumbilical defect. The cystic structure showed preserved vascularization (Figure 4b). Ultrasonographic examination revealed the cystic structure contained anechoic fluid with multiple septations. There were no associated congenital defects. Surgery was performed within the first 24 h of life. Initial surgical exploration revealed proximal jejunal atresia with colonic atresia and an extra-abdominal cystic structure containing 90 mL of straw-colored fluid, supplied by midgut mesenteric vessels (Figure 4c); the cystic formation was excised and jejunocolonic anastomosis was performed. Morphological analysis of the cystic formation revealed colonic tissue architecture (Figure 4d). There were no postoperative complications. The patient has SBS, requiring subsequent management in the gastroenterology department.

### 3.5. Case 5

**Figure 5:**
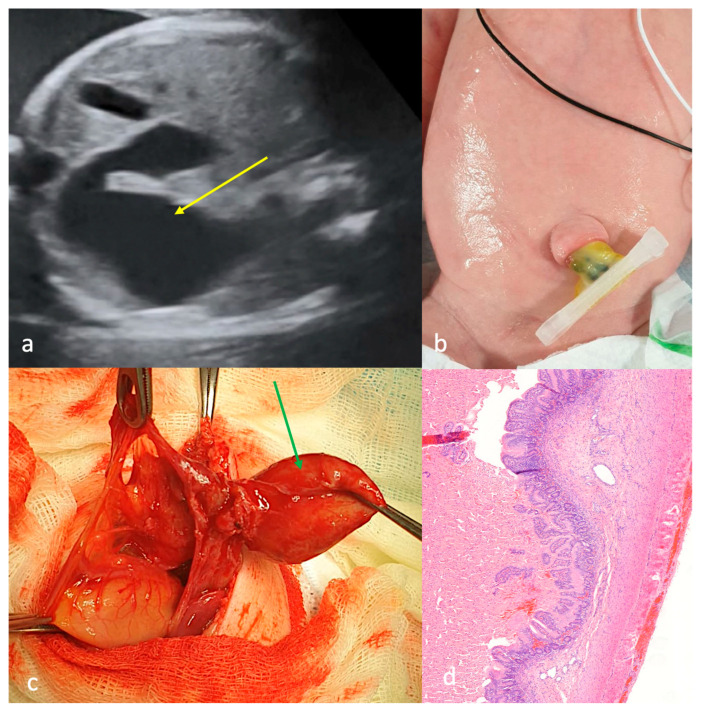
Case 5 with CGS type D. (**a**)—ultrasound screening at 30 weeks of gestation: dilated loop in the abdominal cavity (yellow arrow); (**b**)—appearance after birth: no defect in the anterior abdominal wall; (**c**)—operation on the first day of life: cystic formation with intestinal wall and mesentery (green arrow); (**d**)—morphological structure of the cystic formation wall: structure of the small intestine wall, including all its layers (magnification ×200).

Gastrochisis was diagnosed during an ultrasound examination in the first trimester. A complete absence of intestinal loops in the amniotic cavity, with signs of intestinal obstruction, was detected at 16 weeks of gestation (Figure 5a). The patient showed neither an anterior abdominal wall defect nor eviscerated intestinal loops (Figure 5b). Ultrasonographic examination of the abdominal cavity revealed a cystic structure containing anechoic fluid with multiple septations. There were no associated congenital defects. The surgery was performed within the first 24 h of life. Initial surgical exploration revealed proximal jejunal atresia with colonic atresia and an intra-abdominal cystic mass (3.0 × 2.0 cm) exhibiting the characteristic bowel wall with associated mesenteric attachment (Figure 5c); the cystic formation was excised and jejunocolic anastomosis was performed. Morphological analysis of the cystic formation revealed jejunal epithelial lining of the cyst wall (Figure 5d). There were no postoperative complications, but the patient subsequently had to be treated in the gastroenterology department due to short bowel syndrome (SBS).

General data on the patients is given in Table 1.

## 4. Discussion

We present a case series of five patients with closing/closed gastroschisis (CGS) managed at our clinic over a two-year period. Each case demonstrated unique antenatal developmental patterns and anatomical characteristics of the congenital defect.

Currently, no consensus exists among pediatric surgeons and obstetricians regarding antenatal management strategies for this complex form of gastroschisis [1,3,4]. In our case series, only two patients received an accurate antenatal diagnosis of CGS, confirmed by the absence of viable intestinal loops in the amniotic cavity at 16 and 27 weeks of gestation. The diagnostic hallmark of complex gastroschisis during the antenatal period remains sonographic signs of intestinal obstruction, which were present in all five cases. Notably, three patients developed signs of obstruction only after 30 weeks’ gestation, including two who postnatally demonstrated viable intestinal loops (CGS type B). In contrast, patients with antenatal necrosis of eviscerated intestinal loops (CGS types C and D) demonstrated an earlier onset of intestinal obstruction signs, detectable at 16 and 19 weeks of gestation. Previous reports documented two cases of CGS type D exhibiting second-trimester (20–22 weeks of gestation) sonographic evidence of intestinal obstruction, accompanied by both an absence of anterior abdominal wall defects and complete blood supply compromise of eviscerated intestinal loops [15,17]. These findings suggest a potential association between early-onset fetal intestinal obstruction and subsequent development of significant vascular compromise in eviscerated intestines in patients with CGS. However, validation of this correlation requires larger cohort studies with systematic documentation of serial ultrasonographic findings across gestational ages. Notably, our literature review revealed no published data characterizing the temporal progression of antenatal changes in CGS type B, which limits comprehensive comparative analysis.

Associated congenital anomalies were uncommon in our CGS cohort, consistent with existing literature. Four of five patients had no additional malformations. The exception was Case 1 (CGS type B), in whom type IIIA jejunal atresia was identified during second-stage surgery. This finding explains why the small cluster of eviscerated intestinal loops contained no meconium at birth in Case 1. This contrasted with Case 2, who had the same type B CGS but whose eviscerated intestinal loops were tightly packed with contents. To our knowledge, there are no reports of patients with CGS type B and associated true small bowel atresia. We believe this is the first report of such a case, which demonstrates that a small volume of eviscerated loops in CGS type B may indicate associated proximal atresia.

The presence of a cystic mass with a viable intestinal wall in two patients (Cases 4 and 5) is noteworthy. In Case 4, this structure emerged only at the 35th week of gestation and was eviscerated through a narrow abdominal wall defect. This specific anatomy has not been previously described in CGS patients. In Case 5, who had a completely closed abdominal wall defect, a similar but smaller cystic formation was located intraabdominally and was not diagnosed prenatally. The pathogenesis of these “enterocysts” in CGS patients, whether intra- or extra-abdominal, remains unclear and warrants further investigation.

In two patients with type B CGS (Cases 1 and 2), staged surgical treatment successfully preserved the entire gastrointestinal tract. The first-stage procedure should involve creating an enterostomy at the site of proximal atresia/stenosis and reducing the eviscerated mass into the abdominal cavity in patients with type B CGS. The decision to avoid primary anastomosis was based on the technical challenges and elevated risk of complications, given the concurrent chemical peritonitis caused by prolonged contact with amniotic fluid and the effects of chronic antenatal ischemia. This approach is supported by the experience of colleagues in treating eight patients with type B CGS [18]. In their initial four patients, the eviscerated mass was resected due to uncertain viability; however, histology revealed healthy intestinal tissue beneath a fibrinous coat. This finding prompted a change in their surgical strategy for the next four patients. The revised approach involved initial enterostomy formation and reduction of the bowel loop complex into the abdomen. After six weeks, a second operation allowed sufficient assessment of all intestinal sections, so that in three of the four cases the entire gastrointestinal tract could be preserved.

We performed the second stage of surgical treatment in patients with type B CGS one month after the first stage, in accordance with current experience [18,19]. In both cases, a 30–40 day interval was sufficient for the complete dissolution of the fibrinous sheath covering the previously eviscerated intestinal loops. However, it is worth noting that the condition of the intestinal loops was less satisfactory in Case 2, who underwent the second procedure 12 days earlier than in Case 1.

The high incidence of short bowel syndrome (SBS) (13–27%) and mortality (10–83%) in CGS patients highlights the need for further research into its etiopathogenesis [3,15,20]. In our series, 3 of 5 (60%) patients developed SBS postoperatively and remain dependent on partial parenteral nutrition.

Table 2 summarizes the key findings from the literature review.

**Figure 6:**
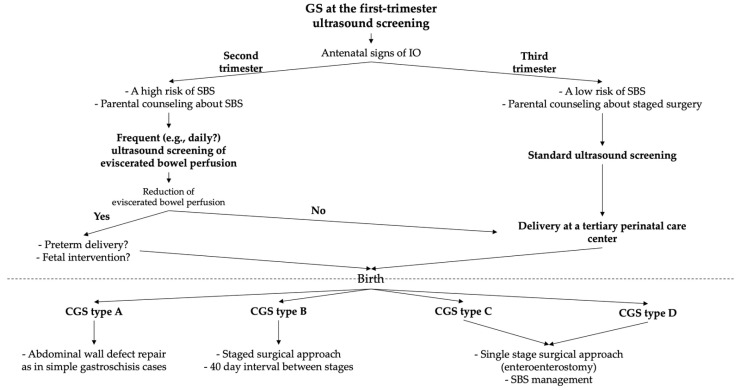
A potential management protocol for patients with suspected CGS. GS—gastroschisis; CGS—closing/closed gastroschisis; IO—intestinal obstruction; SBS—short bowel syndrome.

The precise etiology, pathogenesis, and timing of antenatal defect closure in CGS remain poorly understood. This knowledge gap has led to considerable variations in clinical practice regarding optimal antenatal monitoring protocols and the timing and method of delivery, and also poses a challenge for establishing evidence-based indications for fetal surgical intervention, when advances in fetal medicine and neonatal resuscitation make such approaches feasible. We propose for discussion a potential management protocol for patients with suspected CGS (Figure 6).

Our study has several limitations that should be acknowledged. A significant limitation is the small cohort of patients with this rare pathology, which precludes the formation of statistically robust conclusions. Additionally, the absence of published data describing the temporal progression of antenatal changes and surgical management according to CGS types using the Perrone classification limits the possibility of a comprehensive comparative analysis.

## 5. Conclusions

In summary, this work demonstrates that antenatal diagnosis of closing/closed gastroschisis (CGS) is feasible. The appearance of sonographic signs of intestinal obstruction in the second trimester may be a predictor for a high risk of subsequent significant vascular compromise of the eviscerated bowel, potentially leading to more severe types of CGS (C and D). To better understand this correlation between the timing of complicated gastroschisis onset and the postnatal CGS type, further experience and multi-center studies utilizing a unified classification are essential. Such research could enable the development of clear guidelines for intensified ultrasound screening, timing of early delivery, and even the consideration of fetal surgery to prevent extensive intestinal loss as advancements in fetal therapy and neonatal intensive care evolve to support such interventions.

Consequently, improving outcomes for patients with closing/closed gastroschisis necessitates a primary research focus on understanding its underlying etiopathogenesis and the antenatal processes that drive its progression.

## Figures and Tables

**Table 1:** Summary. CGS—closing/closed gastroschisis; IO—intestinal obstruction; SBS—short bowel syndrome.

	Case 1	Case 2	Case 3	Case 4	Case 5
Type of CGS	B	B	C	C (?)	D
CGS diagnosed antenatally (gestation week)	-	-	27	-	16
IO appearance antenatally (gestation week)	31	31	19	32	16
Determination of non-viability of eviscerated intestinal loops antenatally (gestation week)	-	-	27	-	16
Gestation week at time of birth	35	38	36	35	35
Type of delivery	natural	c-section	c-section	c-section	natural
Weight after birth (in grams)	2310	3850	2620	2750	2400
Associated malformations	Type IIIA jejunal atresia	-	-	-	-
Stages of surgical treatment (days of life)	2 (1; 44)	3 (1; 32; 93)	1 (1)	1 (1)	1 (1)
Postoperative complications	-	Sepsis	-	-	-
Preserved parts of the intestine	All	All	Duodenum; 15 cm of jejunum; half of colon	Duodenum; 20 cm of jejunum; ¼ of colon	Duodenum; 5 cm of jejunum; half of colon
Outcome (age)	Healthy (2 years old)	Lethal	SBS (2 years old)	SBS (2.5 years old)	SBS (2.5 years old)
Necessity of parenteral nutrition	No	-	Partially	Partially	Partially

**Table 2:** Summarized literature review. CGS—closing/closed gastroschisis; TPN—total parenteral nutrition; SBS—short bowel syndrome.

Author (Year)	CGS Cases (*n*)	Antenatal Features	Surgical Approach	Outcomes
Dennison (2016) [15]	1 (+ review of 13 cases)	Second-trimester intestinal obstruction signs; absence of eviscerated bowel	Jejunoileal anastomosis	85–100% mortality; SBS in survivors
Verma et al. (2025) [16]	6	Not systematically reported	Ileocolic and jejunoileal anastomosis	16.7% mortality; all survivors had SBS
Ponce et al. (2018) [17]	1	Intestinal obstruction at 20–22 weeks; progressive disappearance of eviscerated loops	Jejunocolic anastomosis	Short bowel syndrome; parenteral nutrition-dependent
Estrada et al. (2008) [18]	8	Not systematically reported	Initial 4 patients—resection of viable bowel under fibrinous coat and jejunocolic anastomosis; Next 4 patients—staged: initial enterostomy + rerouting of eviscerated bowel into the abdominal cavity; second look at 6 weeks	3/4 patients had full intestinal preservation after protocol change
Field et al. (2021) [19]	11	Progressive defect narrowing (≤8 mm) after 30 weeks of gestation in 62%	Patients with closing gastroschisis and no obviously necrotic bowel were treated with parking of the extruded bowel at initial presentation	Closing gastroschisis had longer times of TPN dependence and lengths of hospital stay than the complicated, non-closing cohort

## Data Availability

In accordance with data sharing guidelines and to facilitate transparency in research, the data upon which this study was based are available upon request. The data are not publicly available due to privacy and ethical restrictions.

## Abbreviations

The following abbreviations are used in this manuscript:

GS	Gastroschisis
CGS	Closing/closed gastroschisis
IO	Intestinal obstruction
SBS	Short bowel syndrome
